# Screening and analysis of acetyl-cholinesterase (AChE) inhibitors in the context of Alzheimer's disease

**DOI:** 10.6026/97320630014414

**Published:** 2018-08-31

**Authors:** Mohd. Babu Khan, Bhagath Kumar Palaka, Tuleshwori Devi Sapam, Naidu Subbarao, Dinakara Rao Ampasala

**Affiliations:** 1 Centre for Bioinformatics, School of Life Sciences, Pondicherry University, Puducherry-605014, India; 2 School of Computational and Integrative Sciences, Jawaharlal Nehru University, New Delhi-110067, India

**Keywords:** AChE, CNS-BBB database, Virtual Screening, MD simulations, Binding free energy, FEL analysis

## Abstract

Acetyl-cholinesterase enzyme (AChE) is a known target for identifying potential inhibitors against Alzheimer diseases (AD). Therefore, it is of interest to screen AChE with the CNS-BBB database. An AChE enzyme is a member of hydrolase family is activated by acetylcholine (ACh), so, targeting the AChE enzyme with the potential inhibitor may block the binding of the ACh. In this study we carried out virtual screening of drug-like molecules from Chemical Diversity Database particularly CNS-BBB compounds, to identify potential inhibitors using Glide docking program. Top ranking ten compounds, which have lower Glide Score when compared to known drugs (Tacrine and Galantamine) for AChE. For top three molecules MD simulation was carried out and calculated binding free energy. We report the best binding compounds with AChE compared to known drugs (Taine and Galantamine) for AD. We further document the salient features of their molecular interaction with the known target. Three molecules (1-benzyl-3-(2- hydroxyethyl)-N-[2-(3-pyridyl)ethyl]-3-pyrrolidinecarboxamide, N-{3[benzyl(methyl)amino]propyl}-1,5-dimethyl-4-oxo-4,5-dihydro- 1H-pyrrolo[3,2-c]quinoline-2-carboxamide, and 6-chloro-N-[2-(diethylamino)-2-phenylethyl]-4-oxo-4H-chromene-2-carboxamide) have -196.36, -204.27, -214.40 kJ/mol, binding free energy values respectively which are much lower than values calculated for the reference ligands Tacrine and Galantamine having -119.65 and -142.18 kJ/mol respectively. Thus these molecules can be very novel potential inhibitors against AChE involved in Alzheimer's disease.

## Background

Alzheimer's disease (AD) is an old age illness affecting many people over the age of sixty and becoming the 7th leading cause of death in all over the world. Just in the United States alone there is an increasing trend in the number of people being diagnosed with AD, the chance of being diagnosed with Alzheimer's increases exponentially and women's are at a larger risk. As per statistics released from the "2018 Alzheimer's Disease (AD), Facts and Figures" report, AD accounts for all types of dementia nearly 60-70% of the cases and an estimated 5.5 million Americans of all ages have Alzheimer's disease and almost 47.5 million people are living with dementia around the world by this disease and it is estimated by 2050 more than 115 million people will have dementia (https://www.alz.org/documents_custom/ 2016-facts-and-figures.pdf) [1-2]. The early diagnosis and the treatment of AD is now an emerging research field, although presently there is no cure for the disease with the existing anti- Alzheimer's drugs, and only moderately effected treatment is possible. At present study on the AD the disability weight (DW), of this disease in people older than 60 years is greater than other lethal disease as like cardiovascular disease (CVD), Stroke, Cancer, and Muscular skeletal disorders. Cholinesterase is the family of the enzymes that catalysis the neurotransmitter acetylcholine (ACh) through hydrolysis into the choline (Ch) and acetic acid [3]. In AD, acetylcholinesterase (AChE) inhibitors, leading to inhibition of acetylcholine (ACh), breakdown and make a way for disease, several strategies to elaborate the disease on the characteristics of symptoms, although the one of that has been most successful till is "cholinergic hypothesis" strategies. (https://jnnp.bmj.com/content/jnnp/66/2/137) [4], the current FDA approved mostly drugs are follows the cholinergic hypothesis, the ACh deficit is; they try to enhance the ACh level in the diseased brain. So, inhibition of AChE level plays an important role for enhancing the cholinergic transmission in the diseased brain. Currently, available AChEI, such as Tacrine (year, 1993) [5], first FDA approved drug used for AD treatments, another Donepezil (year, 1996) [6], Rivistigmine (year, 2000) [7], and Galantamine (year, 2001) [8], are found moderately effected to treat the level of the diseased brain, but approximately, 40-70% patients are beneficial from AChEI. AChE [E.C. 3.1.1.7] is the found many types of transporting in tissues likewise nerve and muscle, central and peripheral, sensory fibers and motor, and cholinergic and non-cholinergic fibers, the level of activity of AChE is much higher in motor neurons than in sensory neurons [9, 10, 11]. AChE also present in Red blood cells (RBCs) membranes, where is consisting Yt blood group antigen and has similar catalytic properties [12]. More about ACh neurotransmitter as the neuromuscular junction between the skeletal muscle and motor nerve, in the central nervous system (CNS), ACh primarily found in the intravenous and few important long-axon cholinergic pathways identified, and degeneration of this pathway is one of the crucial pathologies and closely related to Alzheimer's diseases (AD) [13, 14]. But non-selectivity of the drugs and limited efficacy, poor bioavailability, other side effects in the periphery, hepato-toxicity are some of the limitations for their success. So, far we need some other effected small molecules, those shown more effected to the elaborate the moderate effect to the best way for the cure of the diseased brain. Thus, the present study was carried out in order to find effective molecules, analysis the interactions mechanism [15]. In about blood brain barrier (BBB), it is a crucial investigation in pharmacological studies under pharmaceutical umbrella in respect to CNS; CNSactive compounds must be crossed BB barrier to interact their specific targets. BBB blocks majority of chemicals do not target the CNS because of unusual side effects [16].

## Methodology

The workflow used in this study is shown in Figure 5.

### Target protein structure

Human acetyl cholinesterase (h-AChE) is a most significant drug target for the therapeutic drugs. Here, we have used high resolution crystal structure of h-AChE a (PDB ID: 4PQE). While preparation of the receptor, all water molecules were deleted and missing hydrogen atoms were added, and prepared via Schrodinger, maestro [17]. It was followed by retrained energy minimization by fixing the residues 543 and remove steric clashes between side chains. Two approved drugs Tacrine & Galantamine used in treatment for Alzheimer's diseases were used as a reference compounds. All other important active site residues were identified using online Coach Server (A Meta- server based approach to protein-ligand binding site prediction (http://zhanklab.ccmb.med.umich.edu/COACH/) [18, 19]. The receptor grid was generated using Glide module [20], of the Schrodinger suite. The receptor grid was generated using the Acyl binding pocket (ABP), Catalytic triad (CT), Peripheral anionic site (PAS), Oxyanionic site, and Anionic sub-site as a grid centre, and the grid boundary was defined between any atoms of ligand and grid boundary is at least 5Å.

### Ligand library preparation

A library of 23,731 CNS_BBB compounds was obtained from the ChemDiv database (http://www.chemdiv.com/cns-bbblibrary/) [21]. CNS-BBB ChemDiv database compound is a known as antioxidant and anti-inflammatory nature, and the 2D structures in Sdf format were downloaded from the ChemDiv database, and created a 3D-Phase database from Phase module of the Schrodinger, phase version 3.2. [22]. In this h-AChE receptor cavity have Catalytic triad (CT), Anionic sub-site (AS), Peripheral Anionic site (PAS), Oxyanion sites, and Acyl binding pocket (ABP) is the main binding sites. Here, docking study is done with the reference compounds of FDA approved anti-Alzheimer's drugs [5, 6, 7, 8]. And other ChEMBL approved under clinical trials compounds also docked the same active/binding site of the receptor, and calculate the binding energy profile of the under clinical trials approved drugs shown in (Table 5 and Figure 6). CNS_BBB data were filtered for Lipinski Rule of Five, using phase module version with few default parameters. Bond orders were assigned and various others states, likewise, tautomer's, stereochemistry, and ring conformations were produced for each input structure. All the structures were minimized using OPLS force field, OPLS force field was used by Glide (grid-based ligand docking with energetics) docking [20].

### Receptor grid generation

A receptor grid was created around the protein binding residues (W86, G121, G122, Y124, E202, S203, A204, W236, F295, F297 Y337, F338, and H447). The reference ligand were sketched by Marvin sketch software and ligand were prepared in LigPrepmodule of Schrodinger suite, which generates all possible states with the neutral pH and generated ionized and tautomer state for the ligands. After protein preparation, Grid generation and LigPrep preparation, ligands were used for molecular docking suite Glide version 9.2 in Schrodinger maestro suite with the extra-precision mode for docking [23].

### Virtual Screening

Here, in this study, we have used CNS-BBB database and followed a cut-off for virtual screening as HTVS-10%, SP-10%, and XP-10%, respectively. The fast & accurate prediction of a ligand tightly and specifically binding to a target protein is a crucial step for computational virtual screening [20].

### Glide Extra-Precision Mode (XP)

The extra-precision (XP) mode of Glide combines a powerful sampling protocol with the use of a custom scoring function designed to identify ligand poses that would be expected to have unfavourable energies, based on well-known principles of physical chemistry. Glide-Score is based on Chem-Score, but includes a steric-clash term and adds buried polar terms devised by Schrodinger to penalize electrostatic mismatches. Experiments were performed using the program GLIDE (Grid-based Ligand Docking) module version 5.6, Schrodinger, LLC, New York, NY, 2011 (Schrodinger Inc.) [23, 24]

### ADME properties

The predicted ADMET properties showed that these compounds could be potent & effective inhibitor against protein ligand interaction b/w Acetyl-cholinesterase and the FDA approved moderately effective drugs. In current study we have identified potential-lead molecules which can be taken for in-vitro studies. The ADME properties of the ligands were predicted using QikProp the compounds prepared were subjected to druglikeness filter. The acceptance criteria of the filter includes Molecular weight (< 500), Q Plog BB (-3.0 to 1.2), Donar HB (0-6), % HOA ( 80% high < 25-poor, > 500-Good), PSA (7-200), Q P log S (-6.5 to 0.5), Metabolism (1-8), Accept HB (2-20), Log P Value o/w (-2.0 to 6.5), CNS (-2 to +2) [25]. All the ligands confirmed to the above mentioned acceptance criteria and they were evaluated for docking using extra precision GLIDE dock module and the results are shown in Table 1, and (Table 3, Table 4, Table 5), respectively.

### Protein –Ligand docking

All the docking calculations were performed using the "Extra Precision (XP)" mode of docking via Schrodinger maestro suite of Glide. A scale factor of 0.8 and partial atomic charge of less than 0.15 was applied to the atoms of both proteins for van der Waals radii. The number of poses generated for each ligand was set criteria to 10,000 and out of them 10 best poses per ligand. The best docked structure from each of the ligand docking calculation was chosen based on XP-Glide Score, Glide energy, and Glide emodel value and interaction of the relative docked complexes were further studied for MD simulations package of GROMACS 5.1.2 and Binding energy (MM-PBSA), calculation, Hydrogen bond Occupancy, with FEL Analyses.

### Molecular dynamics simulations

GROMACS 5.1.2 molecular dynamics package [26] was used to know the structural stability of the selected protein-ligand complexes. All the generated protein structures were processed further on WhatIf server for completing the structures [27], and ligand topology was generated using the PRODRG server [28]. Further, proteins were solvated by SPC216 with Spce-ignh water model in the triclinic-box size of 1.0 nm distance. The bond angles and geometry of the water molecules were constrained with LINCS [29], and SETTLE [30]. The van der Waals and electrostatic long-range interactions were applied by using fast Particle-Mesh Weald electrostatics (PME) [31]. Additional, Parrinello-Rahman [32] method was used to regulate the pressure, whereas modified weak Coupling Bearsden thermostat and V-rescale algorithm were used to regulate the temperature of the system. NVT and NPT were accomplished for 100ps and monitored for their equilibration status. Finally, the system was subjected to 30ns of production MD simulation run with a time frame of 2fs [33]. Binding energy calculation was performed by MM-PBSA [34]. FEL analysis was performed to check the quantification of the trajectory changes. The cosine value less than 0.5 are considered favourable to generate the good plots of FEL analysis [35, 36].

## Results

### Virtual screening

The virtual screening workflow consists of three important steps for good selectivity criteria of the compounds, as first step was high throughput virtual screening (HTVS), which was followed by standard precision (SP) docking & finally extra-precision (XP) docking. The top 10% of the compounds identified in the first step (23,731-HTVS-2373) were selected and moved to the second step of the virtual screening workflow and follow the same 10% for second step for (2,373-SP-237), and once again the same for 10% for (237-XP-23) screening, respectively. Virtual screening against AChE, three top scoring compounds were screened from CNS-BBB data with the higher XP glide score and binding free energy as compared to the known FDA approved Alzheimer's inhibitor drugs selected for further study shown in Table 1.

### Molecular Docking Studies

**Figure 2:**
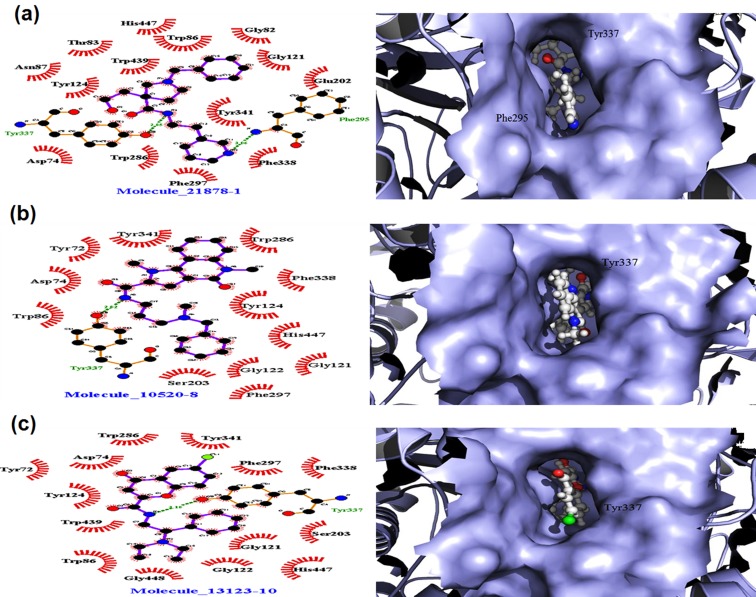
Docking view of the M1 (2A), M8 (2B), and M10 (2C) docked ligand in the binding site of protein. Dotted green lines shows H-bonds in LigPlot view, and ligand in sphere view in the active site cavity of surface view of PyMol.

Among all the docked compounds, three top scoring Molecule21878, Molecule10520 and Molecule13123 showed good interaction and binding energy against human-AChE. In order to understand the binding orientation and non-bonding interaction, that performed well in docking and MD simulations, this study were executed and compared with the binding mode of interaction of the known FDA approved and under trial drugs ligands. Examine of the binding of approved inhibitor ligand shown that Tacrine, and Galantamine (both are well-known FDA approved drugs), have Tyr 133 (2.64 Å) and Ser203 (3.01 Å) interaction in the cavity of the human-AChE enzyme, and docking amino acid interactions between ligand and receptor shown in Figure 2A, 2B and 2C.

### Post docking analysis

The highest XP Glide score of -15.19 kcal/mol, -13.70 kcal/mol, and -13.57 kcal/mol were found for molecule21878 (M1), molecule10520 (M8), and molecule13123 (M10), respectively shown in Table 1. Whereas, other compounds are also showed good XP Glide score value with AChE enzymes, but the binding free energies predictions from MD simulations were also high for these top three compounds. All the hits compounds were docked into the active binding site of the protein and the top scoring pose for ligand was saved for further analysis.

### Binding mode of screened compounds

#### Molecule 21878 (M1)

This compound bind within the active site of AChE specially in Acyl binding pocket (ABP) and Peripheral anionic site (PAS) with a XP Glide score -15.19 kcal/mol and binding free energy -196.36 kJ/mol (Table 1 & Table 2). It formed three hydrogen bonds with the amino acids Asn87, Phe295 and Tyr337 with the distance of 3.06Å and 2.85 Å respectively shown in Figure 2A. Total 14 hydrophobic & 69 Non-bonded interactions were exhibited by the Asp74, Gly82, Thr83, Trp86, Gly121, Tyr124, Glu202, Trp296, Phe297, Phe338, Tyr341, Trp439, and His447. Mostly, hydrophobic interactions were observed in ABP and PAS region with Oxyanionic site, anionic sites and catalytic triads.

#### Molecule 10520 (M8)

The binding free energy from this compound was -204.27 kJ/mol shown in Table 2, and XP Glide score value was -13.70 kcal/mol. One hydrogen bond was observed with Tyr337 from ABP site with the distance of 2.82 Å shown in Figure 2B. In addition, 12 hydrophobic and 92 Nonbonded interactions were evolved in stabilizing the complex. Amino acids Tyr72, Asp74, Trp86, Gly121, Gly122, Trp124, Ser203, Trp286, Phe297, Phe338, and Tyr341 were involved in several hydrophobic interactions within ABP, catalytic triad, PAS and Oxyanion site shown in Figure 2B.

#### Molecule 13123 (M10)

This compound made a complex with XP Glide score value of -13.57 kcal/mol, and has a binding free energy -214.40 kJ/mol calculated from g_MMPBSA shown in Table 2. This compound was found to have one H-bond with Tyr337 has distance with 3.18 Å shown in Figure 2C. Moreover, complex was also stabilized with 14 hydrophobic and 76 Nonbonded interactions with residues Tyr72, Asp74, Trp86, Gly121, Gly122, Tyr124, Ser203, Trp286, phe297, Phe338, Tyr341, Trp439, and His447. Compounds ID & IUPAC name list with detailed shown in (Table 6), and all combined docking related poses of M1, M8 and M10 are shown in (Figure 7, Figure 8, Figure 9) respectively.

### Molecular dynamics and post-dynamic analysis

**Figure 3:**
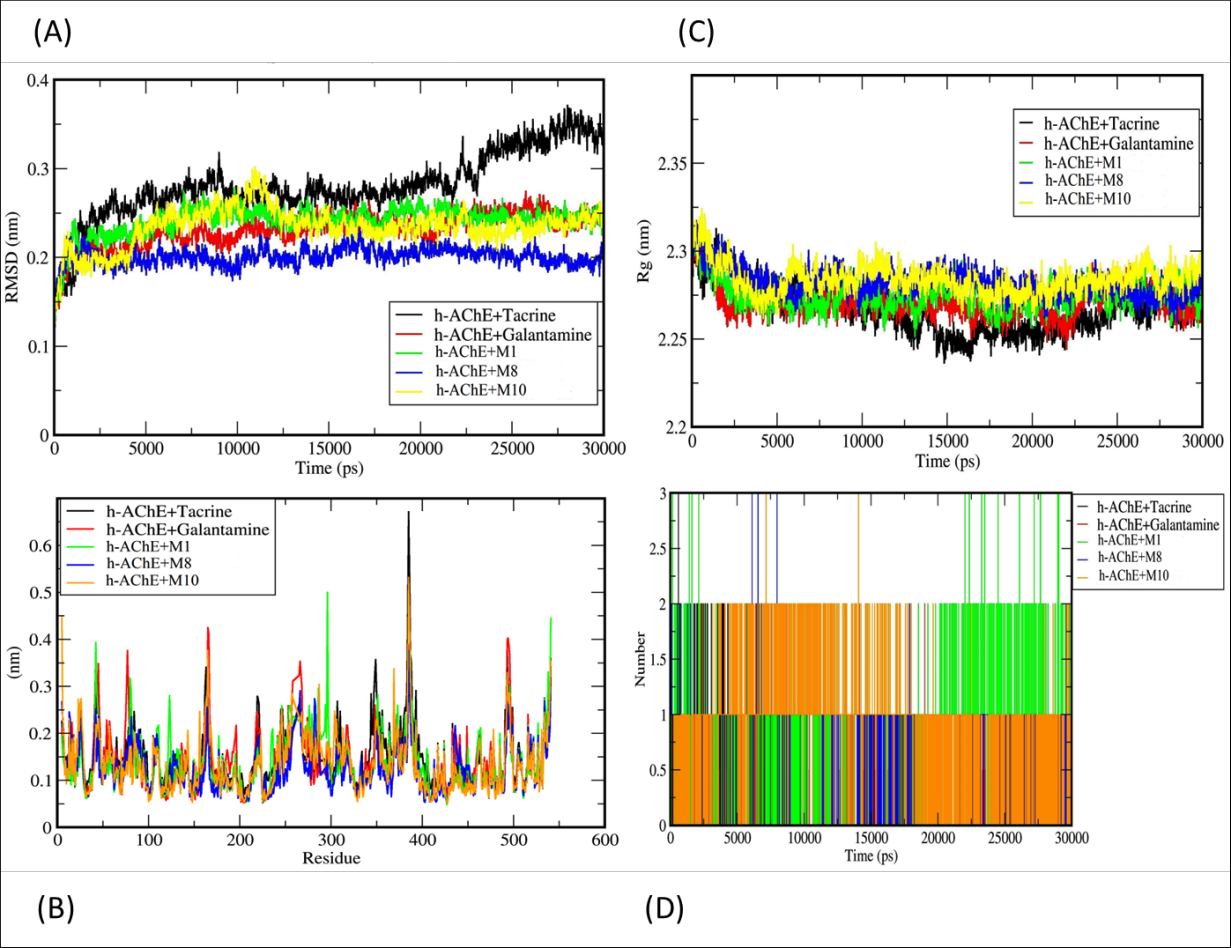
Molecular dynamics simulations Trajectory-graph for (A) RMSD, (B) RMSF, Gyrate (C), and H-bonds (D), for all three hit compounds along with both references (Tacrine & Galantamine). The time period scale used is 30ns.

On the basis of best-docked ligand enzyme complexes that resulted compounds were them to expose further to MD simulations using the GROMACS 5.1.2 package [33]. And followed the procedure explanations in our computational methodology part of this manuscript. Here, we examine the postdynamic nature of the how ligand interacted with the h-AChE target receptor within a range as of 5Å, depicted by H-bonds and Hydrophobic interactions using LigPlot (v.1.4.5) and PyMol version of Schrodinger (v.1.3). And other graphs were prepared by used Xmgrace tools. In our study, MD simulations of 30ns were performed for the top ten, score ligand-complexes shown in Figure 3, to ensure the stability of the ligand within the h-AChE active site as CAS, PAS and Oxyanions sites. Here, interestingly for all the ligand-enzyme complexes the average RMSD values were coming to the range between 1.5Å to 3.50Å, in addition, the gyrate or radius of gyration is showed good compactness in the range between 2.32 nm to 2.27 nm, and also RMSF graph shows well compare to the reference ligands, fluctuations is not much observed in hit complexes, H-bonds shown in Figure 3D, and H-bonds with H-bond-Occupancy shown in (Table 4). We took our study ahead by obtaining a top three CNS-BBB chemDiv compounds out of 23,731, through virtual screening, these are identified leads have the propensity to be considered as a potential Human-AChE anti-Alzheimer's compounds. All the insilico information gained from this study could be shed light on the new series of lead compounds as potential h-AChE inhibitors.

### Molecular dynamic simulations analysis

A 30ns of molecular dynamic simulations was performed for each complex to access the stability of the enzyme-ligand complexes shown in Figure 3A, the root mean square deviation (RMSD) value of the AChE-Ligand complex over the simulation of time. RMSD plot shown stable backbone trajectories of M1 (average b/w 2.25 Å -2.30 Å), M8 (average b/w 1.5 Å - 2.0 Å), and M10 (average b/w 2.20 Å - 2.30 Å ) as compared to reference h-AChE+Tacrine (R1) (average 3.5 Å). Whereas binding of second reference h-AChE+Galantamine (R2) (average 2.48 Å), have shown slightly stable trajectories comparatively than R1. Here, it can be concluded that these molecules, M1, M8, and M10 are the most preferable compounds for h-AChE inhibitors. In the root mean square fluctuations (RMSF) profile, five to six peaks observed high, but one high peak was found between the residues 380-390 where it was observed that only M8 restricted little movement, while in all other ligand and R1 and R2 ( reference ligand) complexes, high fluctuations was observed in this region shown in Figure 3B. Here, our results suggested that h-AChE enzyme and selected hit compounds were able to maintain their structural integrity during the simulations. In the radius of gyration of an object describes its dimension, calculated as root mean square distance between centre of gravity and its ends. Radius of gyration is indicative of level of compaction in the structures. In context of Radius (Rg) value is measure of the compactness of a protein complex, radius is a measurement of the stability of the folded protein. Radius of the initial starting structure was 2.32 nm and the value goes too decreased to 2.27 nm at the end of the 30 ns and MD simulations showed that protein ligand complexes were stable and well folded shown in Figure 3C. In h-bond analyses profile of h-AChE docked systems showed consistent h-bond trajectory in M1, M8, and M10 average 2-5 h-bonds were found throughout the trajectory, where in case of reference R1, one h-bond found throughout the time scale shown in Figure 3D. On the basis of MD simulation studies, among the three screened compounds, compound M10 have shown highest binding free energy against h-AChE, with -214.40 kJ/mol. Shown in Table 2.

### Free Energy Land Scape (FEL) Analysis

**Figure 4:**
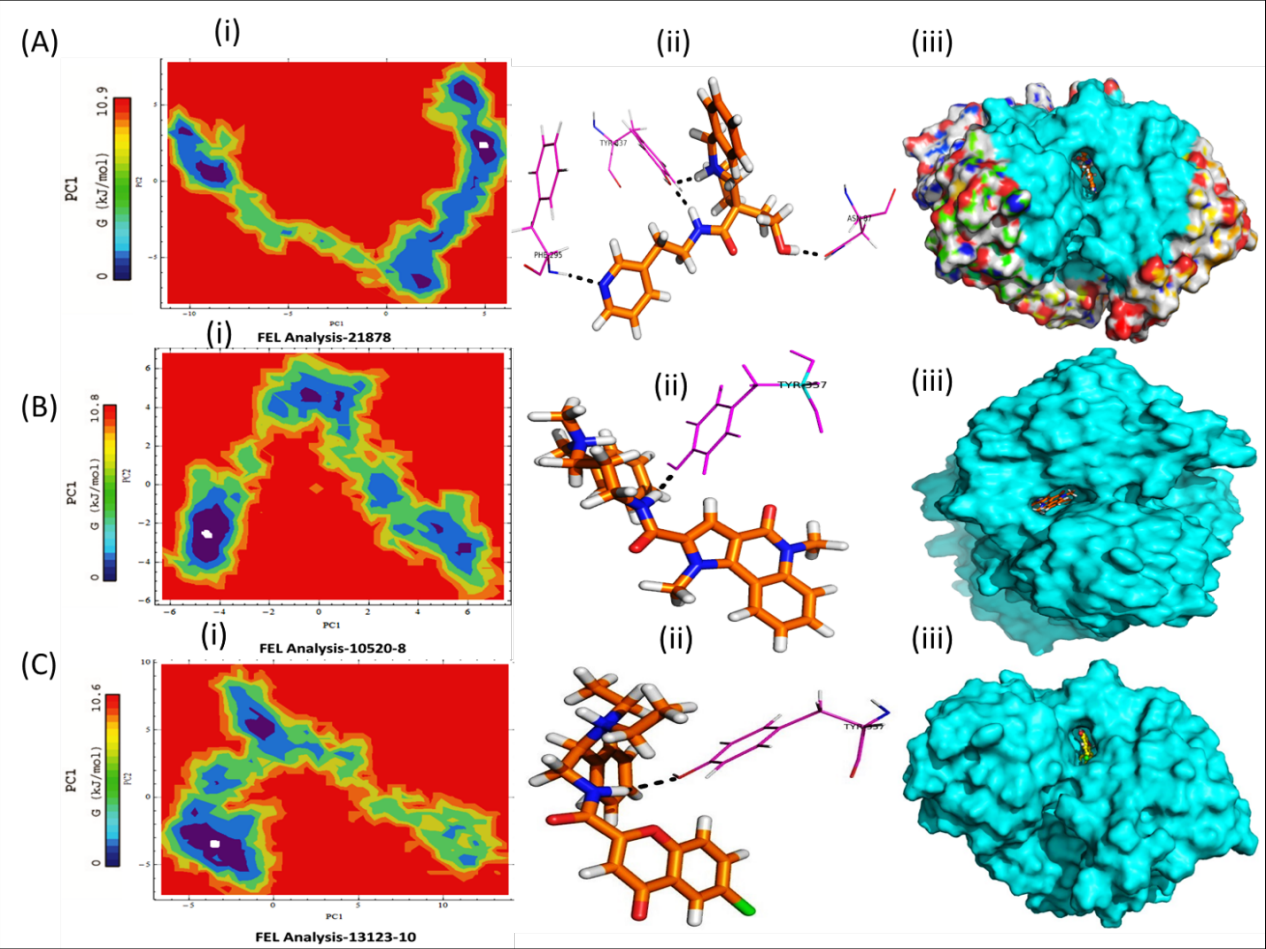
Two dimensional FEL Plots of top three scoring ChemDiv hits ligands (A) M1, (B) M8, and (C) M10 retrieved at 21, 10 and 13ns, respectively, and Ligand Interaction with residues (i, ii, and iii) along with surface view poses, respectively.

**Figure 5:**
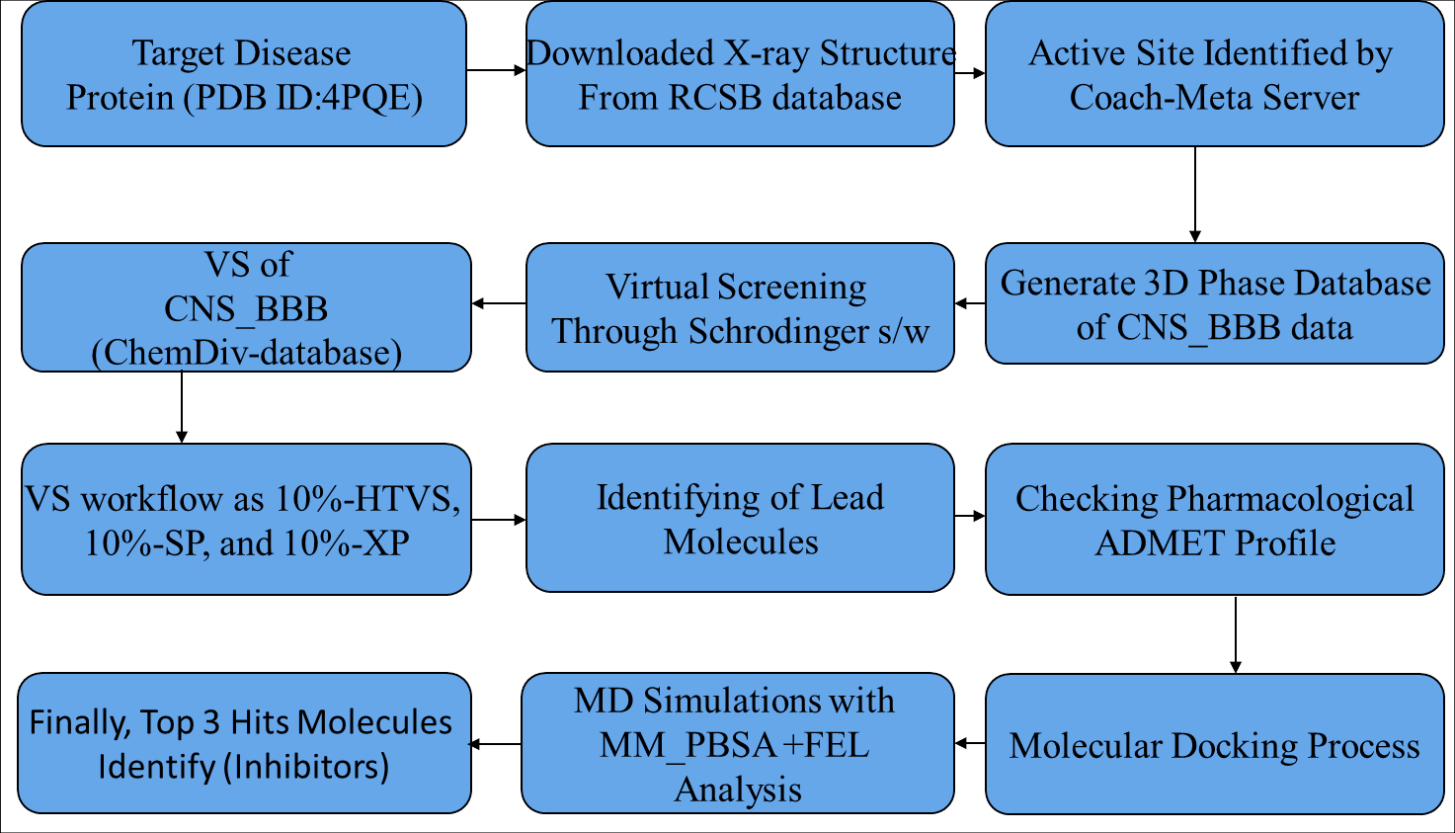
Flowchart for the study from start to end.

**Figure 6:**
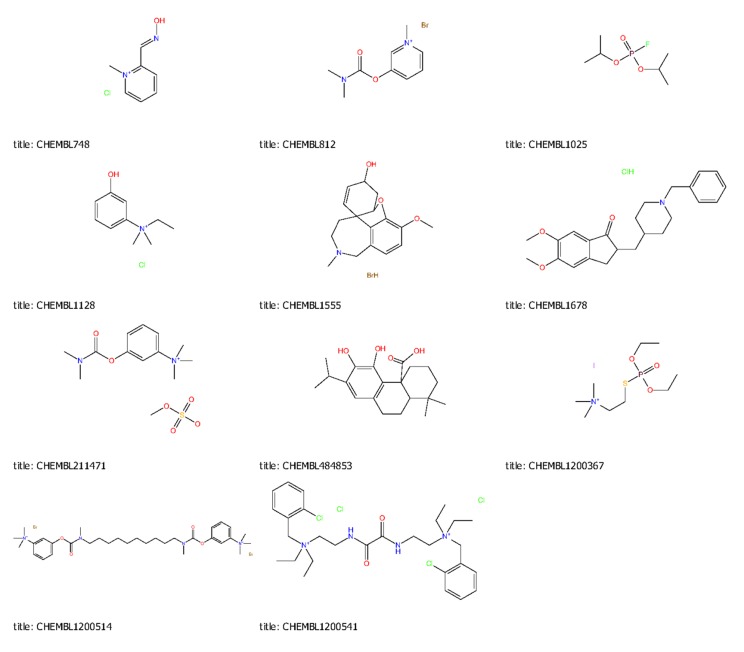
2D-Structure of CHEMBL approved clinical trial drugs.

**Figure 7:**
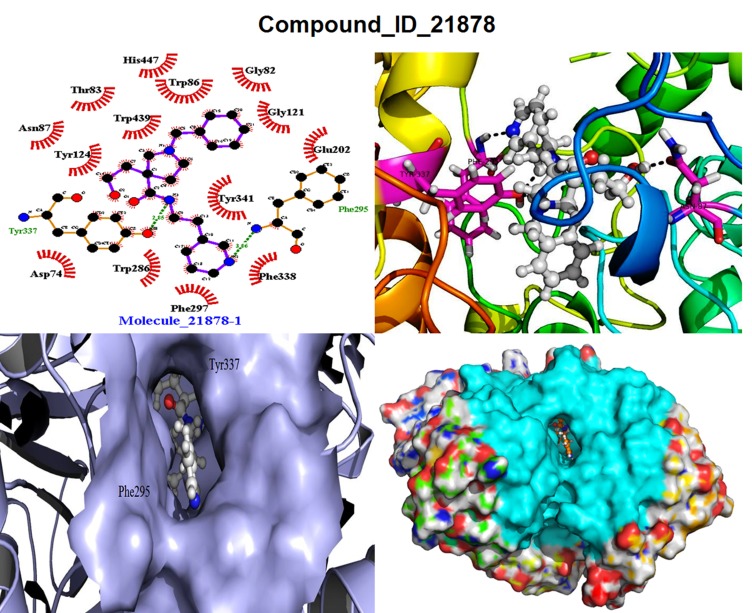
Docking pose view of the M1 ligands and their amino acid interactions in the active/binding site of the h-AChE enzyme, views are in LigPlot, Ligand with receptor, Ligand in active site of the gorge in surface view in (lavender-color) & full surface view in (cyan-color).

**Figure 8:**
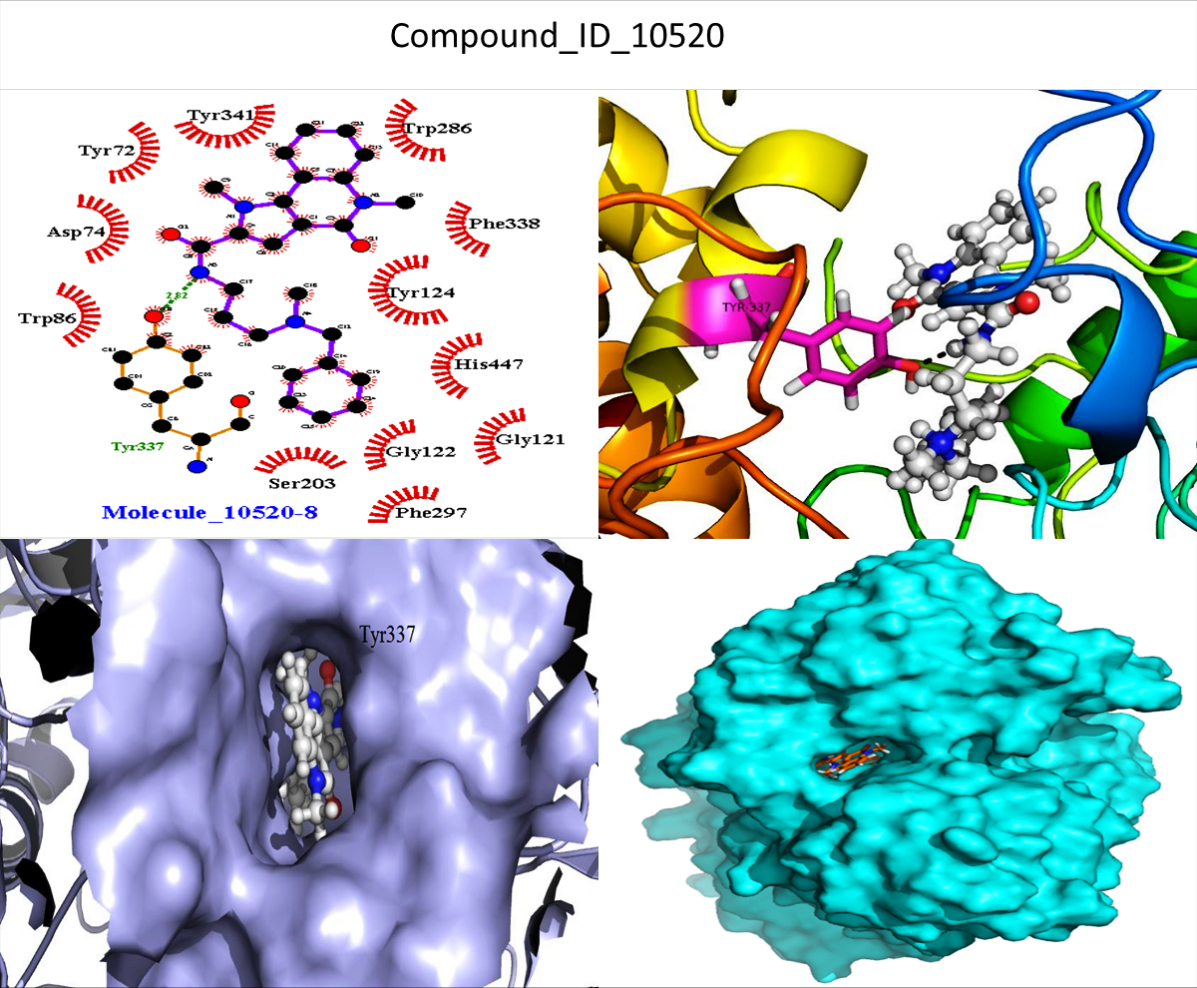
Docking pose view of the M8 ligands and their amino acid interactions in the active/binding site of the h-AChE enzyme, views are in LigPlot, Ligand with receptor, Ligand in active site of the gorge in surface view in (lavender-color) & full surface view in (cyan-color).

**Figure 9:**
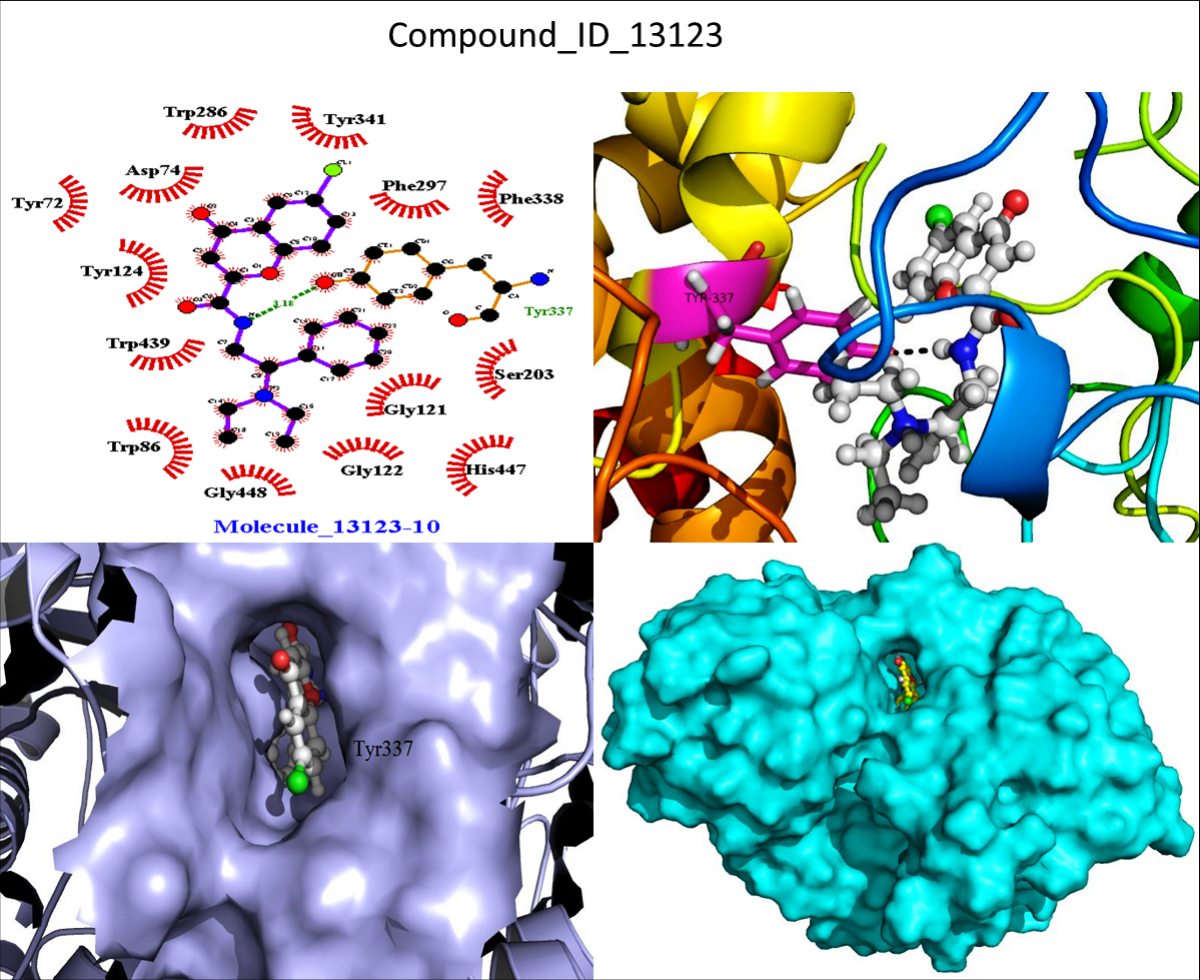
Docking pose view of the M10 ligands and their amino acid interactions in the active/binding site of the h-AChE enzyme, views are in LigPlot, Ligand with receptor, Ligand in active site of the gorge in surface view in (lavender-color) & full surface view in (cyan-color).

In free energy land scape analyses, all the possible state of minimum energy conformations that a protein can adopt during simulation has been studied. This analysis is based on Gibbs free energy. In the lowest energy conformations of Tacrine, and Galantamine (as reference ligand inhibitors) and top three binding energy ligated complexes M1, M8 and, M10 were retrieved and their interactions were analysed. Where, Tacrine- AChE, and Galantamine-AChE maintained its H-bonds and ∏-∏ interactions with Ser125 H-bond side chain in Tacrine, and Trp86 have two ∏-∏ interactions, and in Galantamine Tyr133 and Ser203 have H-bonds, respectively. Moreover, hydrophobic interaction with Asp74, Asn87, Glu202 and His447. Further, in M1 residues Asn87, Phe295, and Tyr337 maintained H-bonds with Trp86 have one ∏-∏ interactions, and other formations like hydrophobic with Thr83, Glu203, and His447. In M8, residues Tyr337 maintained Hbond, and Trp286 with Tyr341 have ∏-∏ stacking interactions, also Asp74, Gly120, Gly121, and Glu203 were involved in hydrophobic interaction, and finally M10, residue Tyr337 shows H-bond interaction with Tyr124 have one ∏-∏ stacking interactions, and Asp74, Gly121, Gly122, Gly448, Ser203 and His447 shows hydrophobic interaction observed shown in Figure 4. Thus, the FEL analysis conveyed the importance of residues Asn87, Ser125, Phe295, and Tyr337 are forming hydrogen bonds in the binding site of the gorge with Trp86, Tyr124, Trp286, and Tyr314 shows ∏-∏ stacking interactions. All the figures of receptor-ligand complexes by depicted by PyMol v.1.3 [37], and LigPlot v.1.4.5 [38].

## Discussion

Based on this CNS-BBB, Standard Data Format (SDF) data, provided by the ChemDiv Data base server, finding the top three ligand inhibitors as M1, M8, and M10 shows a good G-Score value, H-Bonds, and ∏-∏ interactions, hydrogen bond occupancy and binding energy with MM-PBSA, Free energy land scape, and residue Tyr337 is playing an important role interacting to all top hits compounds with the CAS and PAS active site, and hydrogen bond occupancy shows a good % (percentage) of H-bonds contributions shown in (Table 4) in interaction analysis by MD simulation studies. In context of the Tacrine as Cognex (year, 1993), was the first FDA approved AChEI, but unfortunately, it has toxicity towards the liver etc. [5], others like Donepezil as Aricept (year, 1996), [6] second anti-Alzheimer's drug, Rivistigmine as Exelon (year, 2000), [7], Galantamine as Nivalin (year, 2001), [8], all are based on cholinergic hypothesis and Memantine was approved (year, 2003), and it is based on amyloid hypothesis, since then no new treatments have been developed for Alzheimer's. Glide score of the top three ligands M1, M8, and M10 are -15.19 kcal/mol, -13.70 kcal/mol, and -13.57 kcal/mol, respectively. While binding free energies (MM-PBSA) for reference ligand Tacrine is -119.65 kJ/mol, Galanatamine is - 142.18 kJ/mol, and M1 have -196.36 kJ/mol, M8 have -204.27 kJ/mol, and M10 have -214.40 kJ/mol, respectively a good score value of free energy, comparatively to the reference ligand, and follows the Lipinski rule of five criteria, so all of them indicate that they can be potential active inhibitors. In the light of the MD simulations results of the Binding free energy calculations, hydrogen bonds, ∏-∏ stacking and physiochemical properties, we conclude that the ligand M1, M8, and M10 to be a potential h- AChE inhibitors.

## Conclusion

We used virtual screening, molecular docking and, MD simulations analysis, and FEL analysis to identify inhibitors against AChE. Compounds were ranked based on glide score and their binding mode. H-bonds interactions, and Binding free energy calculations with Free energy landscape analyses were also reported. We found that all the top ranked molecules interacted with the catalytic triad, peripheral anions site, Anionic sub-site, Oxyanion site, and Acyl binding pocket, of the h-AChE enzyme region with aromatic amino acid residues as Tyr124, and Tyr337, And Phe295, Asn87 residues are the key residues involved in the binding interactions. We document the top three molecules ligands as M1, M8, and M10, as potential h-AChE inhibitors based on MD simulations, binding free energy and physiochemical properties.

## Conflict of Interest

Authors declare no conflict of interest.

## Figures and Tables

**Table 1:** Protein-ligand interactions for Top hit compounds and referenced FDA approved drugs Tacrine and Galantamine. (NOH: Number of Hydrophobic Interactions, NIB: Number of Non-Bonded Interactions).

S.No	Compound Name & PubChem CID	IUPAC Name	G-Score Kcal/mol	Number of residues, H-Bonds, & ∏-∏ Bonds Interactions	NOH	NIB
R1.	Tacrine CID1935	1,2,3,4-tetrahydroacridin-9-amine	-8.89	2,W86, S125	10	43
R2.	Galantamine CID9651	(4aS, 6R, 8aS)- 5,6,9,10,11,12- hexahydro- 3-methoxy- 11-methyl- 4aH- [1]benzofuro[3a,3,2-ef] [2] benzazepin- 6-ol	-6.09	2,Y133, S203	11	62
1	M21878, CID124077156	1-benzyl-3-(2-hydroxyethyl)-N-[2-(3-pyridyl)ethyl]-3-pyrrolidinecarboxamide	-15.19	3, N87, F295, Y337	14	69
2	M21882, CID124077156	1-benzyl-3-(2-hydroxyethyl)-N-[2-(3-pyridyl)ethyl]-3-pyrrolidinecarboxamide	-14.84	2,F295, Y337	14	70
3	M787, CID20856548	N-(3-azepan-1-ylpropyl)-2-[(7-chloro-1-methyl-2-oxo-1,2-dihydroquinolin-4-yl)thio]acetamide	-14.67	3,D74, F295, Y341	11	67
4	M21880, CID124077156	1-benzyl-3-(2-hydroxyethyl)-N-[2-(3-pyridyl)ethyl]-3-pyrrolidinecarboxamide	-14.58	2,F295, Y337	12	70
5	M21884, CID124077156	1-benzyl-3-(2-hydroxyethyl)-N-[2-(3-pyridyl)ethyl]-3-pyrrolidinecarboxamide	-14.22	2,F295, Y337	12	64
6	M2651, CID6485896	5-({[2-(4-fluorophenyl) ethyl] amino} methyl)-1,3-dimethyl-1,3-dihydro-2H-benzimidazol-2-one	-13.94	1,F295	12	63
7	M2651, CID6485896	5-({[2-(4-fluorophenyl) ethyl] amino} methyl)-1,3-dimethyl-1,3-dihydro-2H-benzimidazol-2-one	-13.83	1,F295	11	76
8	M10520, CID20865255	N-{3-[benzyl(methyl)amino]propyl}-1,5-dimethyl-4-oxo-4,5-dihydro-1H-pyrrolo[3,2-c]quinoline-2-carboxamide	-13.7	1, Y337	12	92
9	M9503, CID46018753	2-{[[2-hydroxy-3-(2-propynyloxy)propyl](isopentyl)amino]methyl}-5-phenylthieno[2,3-d]pyrimidin-4(3H)-one	-13.64	3,Y124, S125, Y337	18	101
10	M13123, CID45155433	6-chloro-N-[2-(diethylamino)-2-phenylethyl]-4-oxo-4H-chromene-2-carboxamide	-13.57	1, Y337	14	76

**Table 2:** Binding free energy of selected ligands against AChE enzyme protein.

Parameters (KJ/mol)	Tacrine	Galantamine	M1	M8	M10
van der Waal energy	-129.14+/14.55	-157.44 +/- 9.36	-226.71+/-10.17	-249.37+/-44.47	-252.75 +/-13.60
Electrostattic energy	-4.76 +/- 4.81	-1.66 +/- 1.74	-18.90 +/- 4.60	-17.55 +/- 6.67	-13.84 +/- 5.47
Polar solvation energy	26.49 +/- 10.94	31.65+/- 9.00	69.73 +/- 10.43	82.62 +/- 10.77	74.75 +/- 14.79
SASA energy	-12.24 +/- 1.45	-14.73+/-0.81	-20.47 +/- 1.00	-19.96 +/- 3.99	-22.55+/- 0.96
Binding energy	-119.65+/14.60	-142.18+/-11.36	-196.36+/-12.19	-204.27+/-50.94	-214.40 +/12.67

**Table 3:** ADMET Property profile of the top ten hits of CNS-BBB of ChemDiv database compounds

Compound Id^a^	Molecular Weight^1^	SASA^2^	QPLogS^3^	QPlog- HERG^4^	QPlog-BB^5^	% HOA^6^	Lipinski's rule of five^7^
M1	353.463	687.395	-2.623	-5.667	-0.689	79.012	0
M2	353.463	688.923	-2.655	-5.697	-0.671	79.542	0
M3	421.984	769.171	-4.25	-5.211	-0.384	83.29	0
M4	353.463	686.182	-2.467	-5.649	-0.719	77.768	0
M5	353.463	688.27	-2.503	-5.682	-0.704	78.257	0
M6	313.374	635.854	-4.673	-6.707	0.106	100	0
M7	313.374	634.414	-4.655	-6.705	0.101	100	0
M8	416.522	777.376	-5.225	-7.703	-0.458	96.477	0
M9	439.571	794.701	-4.39	-7.341	-0.575	95.746	0
M10	398.888	689.205	-4.179	-6.483	-0.319	91.284	0
^a^ChemDiv ID of the compound, ^1^Molecular weight of the molecule, ^2^Surface area, ^3^Predicted aqueous solubility, ^4^Predicted IC_50 _value for blockage							

**Table 4:** H-Bonds Occupancy profile of the referenced ligands and potential compounds from CNS-BBB data of ChemDiv database

Interacting residues	H-bond in FDA drug-h-AChE docking	H-Bond in CNS-BBB docking	% Occupancy of H-Bond in MD simulations			
FDA drug h-AChE MD Simulation	CNS-BBB Compounds		
	M1	M8	M10
D74	No	No	-	-	30	-
N83	No	Yes	-	-	-	-
S125	Yes	No	-	-	-	-
S203	Yes	No	-	-	-	-
W86	Yes	Yes	-	-	-	-
W286	No	No	1.8	30	-	-
F295	No	Yes	-	-	-	50.8
Y124	No	No	2.5		26.5	19.9
Y133	Yes	No	-	-	-	-
Y337	Yes	Yes	12.7	-	-	-
Y341	No	Yes	-	-	-	-

**Table 5:** CHEMBL clinical approved drugs on trials, showed binding Energy profile of five-top compound as referenced: (CHEMBL ID)

Parameters (kJ/mol)	CHEMBL-1200541	CHEMBL-1555	CHEMBL-1678	CHEMBL-211471	CHEMBL-1128
van der Waal energy	-260.10+/- 13.44	-155.13 +/- 6.46	-202.97 +/- 78.47	-157.09 +/- 7.59	-123.37 +/-7.01
Electrostattic energy	-34.22+/- 9.48	-2.57+/- 0.909	-1.07+/- 1.57	-12.9 +/-8.40	-0.14+/- 1.70
Polar solvation energy	118.42+/-15.36	36.29+/-10.09	37.08+/-11.00	50.61+/- 9.99	35.89+/- 5.58
SASA energy	-23.98 +/- 1.278	-15.63+/- 0.86	-19.17+/-7.12	-14.04+/- 0.64	-11.52+/- 0.63
Binding energy	-199.88 +/-14.15	-137.05+/-10.62	-186.13+/-82.56	-133.45+/-11.12	-99.16+/- 9.31

**Table 6:** CNS-BBB, (ChemDiv database) Molecule Name with ID Number, &IUPAC Name

S. No.	Molecule Name	ID NUMBER	Pubchem CID	IUPAC Name
1	M1	S729-0059	124077156	1-benzyl-3-(2-hydroxyethyl)-N-[2-(3-pyridyl)ethyl]-3-pyrrolidinecarboxamide
2	M2	S729-0059	124077156	1-benzyl-3-(2-hydroxyethyl)-N-[2-(3-pyridyl)ethyl]-3-pyrrolidinecarboxamide
3	M3	C463-0344	20856548	N-(3-azepan-1-ylpropyl)-2-[(7-chloro-1-methyl-2-oxo-1,2-dihydroquinolin-4-yl)thio]acetamide
4	M4	S729-0059	124077156	1-benzyl-3-(2-hydroxyethyl)-N-[2-(3-pyridyl)ethyl]-3-pyrrolidinecarboxamide
5	M5	S729-0059	124077156	1-benzyl-3-(2-hydroxyethyl)-N-[2-(3-pyridyl)ethyl]-3-pyrrolidinecarboxamide
6	M6	D272-0723	6485896	5-({[2-(4-fluorophenyl)ethyl]amino}methyl)-1,3-dimethyl-1,3-dihydro-2H-benzimidazol-2-one
7	M7	D272-0723	6485896	5-({[2-(4-fluorophenyl)ethyl]amino}methyl)-1,3-dimethyl-1,3-dihydro-2H-benzimidazol-2-one
8	M8	C593-0453	20865255	N-{3-[benzyl(methyl)amino]propyl}-1,5-dimethyl-4-oxo-4,5-dihydro-1H-pyrrolo[3,2-c]quinoline-2-carboxamide
9	M9	V022-7277	46018753	2-{[[2-hydroxy-3-(2-propynyloxy)propyl](isopentyl)amino]methyl}-5-phenylthieno[2,3-d]pyrimidin-4(3H)-one
10	M10	D491-2213	45155433	6-chloro-N-[2-(diethylamino)-2-phenylethyl]-4-oxo-4H-chromene-2-carboxamide

**Figure 1:**
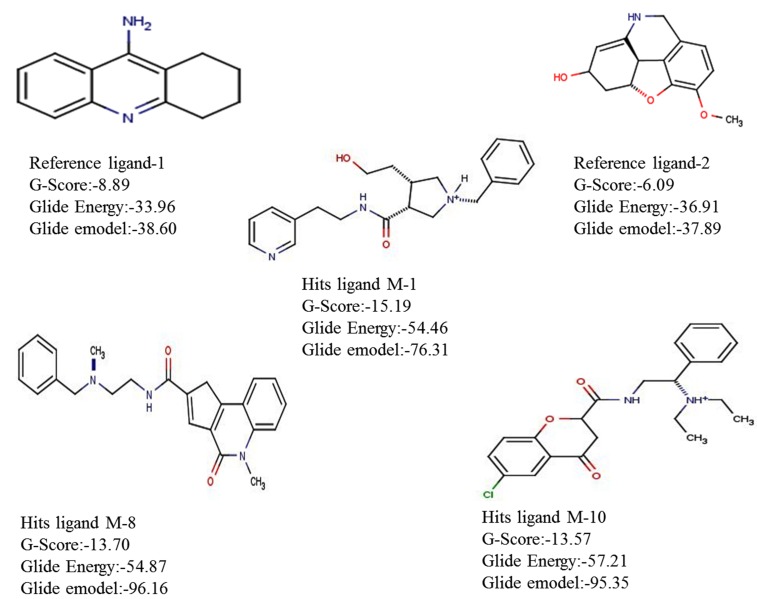
Schematic representation of 2D images of reference ligands structures, Tacrine & Galantamine used in this study & three top hits compounds M1 (1-benzyl-3-(2-hydroxyethyl)-N-[2-(3-pyridyl)ethyl]-3-pyrrolidinecarboxamide),M8(N-{3-[benzyl(methyl) amino] propyl}-1,5-dimethyl-4-oxo-4, 5-dihydro-1H-pyrrolo[3,2-c]quinoline-2-carboxamide), and M10 (6-chloro-N-[2-(diethylamino)-2-phenyl ethyl]-4-oxo-4H-chromene-2-carboxamide),represented, respectively) in molecular docking.

## Abbreviations

AChE-: Acetyl cholinesterase
AD: Alzheimer's Diseases
HTVS: High Throughput Virtual Screening
MDS: Molecular Dynamic Simulations
RMSD: Root Mean Square Deviation
RMSF: Root Mean Square Fluctuations
FEL: Free Energy Land Scape
